# Depletion-of-susceptibles bias in influenza vaccine waning studies: how to ensure robust results

**DOI:** 10.1017/S0950268819001961

**Published:** 2019-11-27

**Authors:** M. Lipsitch, E. Goldstein, G. T. Ray, B. Fireman

**Affiliations:** 1 Department of Epidemiology, Center for Communicable Disease Dynamics, Harvard T.H. Chan School of Public Health, 677 Huntington Avenue, Boston, MA 02115, USA; 2 Department of Immunology and Infectious Diseases, Harvard T.H. Chan School of Public Health, 665 Huntington Avenue, Boston, MA 02115, USA; 3 Division of Research, Kaiser Permanente, 2000 Broadway Oakland, CA 94612, USA

**Keywords:** Epidemiology, influenza, influenza vaccines, statistics, waning

## Abstract

Vaccine effectiveness studies are subject to biases due to depletion-of-persons at risk of infection, or at especially high risk of infection, at different rates from different groups (depletion-of-susceptibles bias), a problem that can also lead to biased estimates of waning effectiveness, including spurious inference of waning when none exists. An alternative study design to identify waning is to study only vaccinated persons, and compare for each day the incidence in persons with earlier or later dates of vaccination to assess waning in vaccine protection as a function of vaccination time (namely whether earlier vaccination would result in lower subsequent protection compared to later vaccination). Prior studies suggested under what conditions this alternative would yield correct estimates of waning. Here we define the depletion-of-susceptibles process formally and show mathematically that for influenza vaccine waning studies, a randomised trial or corresponding observational study that compares incidence at a specific calendar time among individuals vaccinated at different times before the influenza season begins will not be vulnerable to depletion-of-susceptibles bias in its inference of waning as a function of vaccination time under the null hypothesis that none exists, and will – if waning does actually occur – underestimate the extent of waning. Such a design is thus robust in the sense that a finding of waning in that inference framework reflects actual waning of vaccine-induced immunity. We recommend such a design for future studies of waning, whether observational or randomised.

Recent studies on influenza vaccine effectiveness (VE) have suggested that effectiveness declines over the course of one season [1–3]. However, these results have been called into question because inferences of waning may be biased. When there is no waning, some study designs (including the classic test-negative observational design [4, 5] and randomised controlled trials [6–8]) may nonetheless infer waning – measured as a decline in VE as the season progresses. This biased inference is predicted to occur when the vaccine offers ‘leaky’ protection, reducing the probability of infection on exposure by some proportion less than 100%, and either or both of the following conditions holds and is unaccounted for in the analysis[9]: (i) some infections occur unobserved in the study population, such that individuals are infected and (for the season) immune to further infection unbeknownst to the researchers [4, 9]; or (ii) heterogeneity in the population exists and is unaccounted for, such that certain persons are at higher risk of becoming exposed or, if they are exposed, of becoming infected upon exposure for reasons other than their vaccine status, for example due to age, past-season history of infection or vaccination or occupation [3, 6, 7].

If either or both of these conditions hold, then over the course of the season, there will be unobserved reductions in the population at risk (or, for the second, at high risk) in each arm of the trial, and these reductions will be greater in any group that receives less vaccine protection, more moderate in a group that is more protected. In a classic comparison of vaccinated *vs.* unvaccinated persons, this ‘depletion-of-susceptibles’ will reduce the pool of susceptible individuals (and especially of highly susceptible individuals) in the unvaccinated group more than in the vaccinated group, reducing the influenza incidence rate in the unvaccinated group relative to the vaccinated group as time progresses; equivalently, the benefit of the vaccine will appear to wane.

Recently, a novel, cohort variant of the test-negative design (TND), was proposed and implemented that sought to circumvent these sources of bias. This design [3] considered only persons who received influenza vaccine and were subsequently tested for influenza infection. As in the classic TND the vaccine history was compared between those testing positive *vs.* negative for influenza infection, but unlike a classic TND, the time from vaccination to influenza test was the exposure of interest (as the study was limited to those who had received vaccine and later received a test). Relative VE for individuals vaccinated at different time points was estimated as a function of this interval, by estimating – at a specific calendar time (using conditional logistic regression) the odds ratio between influenza test-positive and test-negative participants, as predicted by time of vaccine receipt and other covariates. Crucial to this method is that individuals with different vaccination dates are compared on a fixed calendar date, rather than (as in the classic TND) comparing individuals with different vaccination statuses on different calendar dates. The time from exposure (vaccination) to outcome (infection) is thus measured precisely and not conflated with calendar time. That study estimated approximately 16% waning in relative effectiveness of vaccination for each 28 days earlier a person had been vaccinated [3].

Peer review and a commentary published alongside the study [5] questioned whether this design had eliminated the potential bias associated with depletion-of-susceptibles. Subsequent discussions led to reanalysis of the dataset with restriction to those who had been vaccinated before influenza season, that is, before infections with influenza could differentially deplete susceptible hosts from different time-of-vaccination groups. The result confirmed the finding of the previous analysis [10]. It was shown heuristically and with simulations that the following was true of the revised analysis: under the null hypothesis that vaccine efficacy did not wane, the study would in expectation be unbiased, estimating that indeed there was no waning, or equivalently that VE was equal regardless of the time since vaccination. Under the alternative hypothesis that vaccine protection does wane, simulations showed that differential depletion-of-susceptibles can bias this analysis towards underestimating waning, but not towards overestimating waning and not towards an incorrect finding that VE wanes. By this logic, a study of pre-season vaccinees only which found no waning might be hard to interpret (either truly null, or waning does occur but bias in the design makes it hard to detect), but a finding that waning does occur could not be attributed to these sources of bias. We also note that waning in the latter study design (vaccinated-only), and the analysis of this paper as a whole, is related to the question whether later vaccination is more protective at any moment when a person might be exposed to influenza than earlier vaccination, due to declining effectiveness of the immune response as time passes post-vaccine; we do not consider here a different potential source of waning, which is antigenic change of circulating influenza strains during the course of a season, making a particular immunised person less protected as a new variant becomes more common.

It would be ethical and informative to undertake a randomised controlled trial in which persons intending to be vaccinated are randomised to early or late vaccination, on dates anticipated to precede the start of influenza circulation (e.g. 1 September *vs.* 15 October) and incidence rates or proportions compared between these two arms, as we have proposed elsewhere [10, 11]. Knowing the expected outcomes under various scenarios would facilitate interpretation of such a trial. Meanwhile, it would be valuable to know precisely under what circumstances designs such as the test-negative case-control approach or a cohort-based modification of that approach (as performed in the example described above [10]) would perform in similar ways. For our purposes, a key difference between the classic test-negative case-control design and a prospective observational or randomised cohort design is that the latter designs attempt to track who is at risk for the outcome, for example by censoring people after they have had one influenza test [3] or after they have had one positive test (a typical randomised trial). By contrast, the test-negative case-control design relies on assumptions that the test-negative participants are representative of the population at risk. Because the biases considered in this study come from the unobserved changes in the susceptibility of the at-risk population, these may be subtly different in the different designs, and we consider several different incidence measures below that represent different approaches to tracking who is at risk.

Here, we consider a hypothetical comparison of two groups of persons, those vaccinated early (group E) and those vaccinated later (group L) with the same vaccine. These might be the two arms of a randomised trial, or might represent an idealised comparison in an observational study; the argument of this paper essentially considers discrete comparisons between days of vaccination, and could be extended to a case where continuous variation in time of vaccination occurs. When we compare two groups vaccinated at different times, with the possibility of waning, it becomes interesting to consider how either the earlier vaccinees or the later vaccinees can be subject to greater depletion-of-susceptibles, and thus the bias in estimating waning can go either way. Specifically, if influenza is circulating between the time when group E is vaccinated and the later time when group L is vaccinated, group L may be more depleted by incidence of infection prior to vaccination in that interval. On the other hand, if vaccine protection in fact wanes, then group E may be more depleted than group L on some or all days after both groups have been vaccinated because the protection in group E will have had longer to wane. Thus, in such a scenario – where group L was vaccinated during the influenza season – either group can be get depleted of its susceptibles faster than the other and so the bias may go in either direction. Here we show how this trade-off occurs, and define a condition under which the bias will overstate waning, or will understate waning, or the estimate of waning will be correct. As particular cases, we show that if vaccination of some individuals occurs after influenza season begins, and there is no waning, then the study will erroneously infer waning has occurred as a result of unobserved differential depletion-of-susceptibles between early- and late-vaccinated participants. If there is waning, the estimated extent of waning may be biased in either direction. On the other hand, if individuals are all vaccinated before influenza season starts (so that there is no risk of infection in any participant before they are vaccinated), and if there is no waning, the study will correctly infer that there is no waning (unbiased estimate). If individuals are all vaccinated before influenza season starts, and there is waning, then the degree of waning will be underestimated (and we cannot rule out an erroneous estimate of increased effectiveness with time since vaccination). These results are summarised in Table 1.

**Table 1.** Summary of findings

	No waning occurs (null hypothesis)	Waning occurs (alternative hypothesis)
Include persons vaccinated after influenza season begins	Biased away from the null: Waning erroneously inferred or overstated (claim 2(i))	Biased: Waning may be over- or under-estimated depending on balance of two effects (claim 2(ii))
Restrict analysis to persons vaccinated before start of season	Unbiased: No waning inferred (claim 2(iii))	Waning underestimated and potentially VE can appear to rise with time since vaccination (claim 2(iii))

## Model

We consider a cohort split into groups and subgroups as described below, and describe its progress through an influenza season. We define a season as a period with nonzero influenza incidence, that is the period during the year during which *λ*(*t*) >0, where *λ*(*t*) is the force of infection with influenza, described more fully below. We denote the start of influenza season as *t*_0_. We assume that within a season it is possible to be infected with influenza at most once. We focus on a comparison between groups with two different dates of vaccination, early (E vaccinated at time *t*_E_) and late (L vaccinated at time *t*_L_>*t*_E_). We consider different scenarios where vaccination of these individuals is complete before (*t*_L_<*t*_0_), or not complete before (*t*_L_>*t*_0_), the start of influenza season. We envision a study in which at some time before influenza season, persons are randomised to be vaccinated early or late, or else choose their vaccination date in a way that is not confounded by predictors of the outcome (test-positive influenza). In this study, all participants are vaccinated; the only difference is when. Throughout the analysis we describe expected outcomes, or equivalently outcomes in an arbitrarily large study, neglecting sampling variation; we also neglect all complexities such as loss to follow-up, non-adherence and the like, to focus on the best-case scenario to infer the existence or non-existence of waning. Waning is inferred to have occurred if influenza incidence at time *t* when measurement of influenza incidence takes place is greater in the early than in the late-vaccinated group, or equivalently, the relative efficacy of the vaccine is greater in the late-vaccinated group than in the early one. Note also that this definition restricts attention to *host* biological processes by which an individual's protection from the vaccine on a given day (with the strains circulating then) is less if vaccination occurred longer ago. We define incidence in three alternative ways below, corresponding to three possible targets for estimation in different observational or randomised study designs.

Now, consider a population group *G* (this will take the value either E or L for early or late vaccinees respectively). *G* is further split into *N* subgroups, each with homogeneous exposure to infection and baseline ‘frailty’ (probability of infection given exposure to infection if unvaccinated) (*i* = 1, …, *N*) such that subgroup *G*_*i*_ is a proportion *f*_*i*_ of the population in *G*. Because we envision a large study with no confounding (by randomisation or simply by assumption), the *f*_*i*_ are equal for both groups (E and L). Let *b*_*i*_*λ*(*t*) be the force of infection to unvaccinated individuals still at risk of infection subgroup *i* at time *t*; again, by randomisation, this is equal for the early and late groups. We refer to *b*_*i*_ as the frailty of group *i*, and we arrange the groups in decreasing order of frailty so that *b*_*i*_ >*b*_*i*+1_. Without loss of generality, we define *b*_*i*_ = 1. We allow for the possibility that some persons may be completely immune to influenza infection throughout the season and assign them (if they exist) to the lowest-frailty group (group *G*_*N*_ with a frailty of *b*_*N*_ = 0). Let *θ*_*G*_(*t*) be 1 minus vaccine efficacy in group *G* at time *t* (thus *θ*_*G*_(*t*) = 1 if *t* <*t*_*G*_, where *t*_*G*_ is the time of vaccination in group *G* and *θ*_*G*_(*t*) ⩽ 1 after vaccination, that is when *t* >*t*_*G*_). Thus we assume the vaccine never increases infection risk for any individual; it is at worst ineffective under extreme waning. For simplicity we assume that *θ*_*G*_(*t*_*G*_) = *θ*_*G*_ <1 and *θ*_*G*_(*t*) is non-decreasing with *t* for *t* >*t*_*G*_ and is constant in the case of no waning. Thus, we assume vaccine is most protective immediately after vaccination, and may wane thereafter. Here we define waning to mean a scenario in which on a particular day, an individual vaccinated longer ago is less protected against infection with the currently circulating strains than had they been vaccinated more recently. We assume that vaccine efficacy, and equivalently *θ*_*G*_(*t*), is the same for all subgroups *G*_*i*_ within *G*; this assumption may be loosened but is kept for the sake of clearer exposition in the proofs.

Let 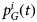 be the proportion of persons in subgroup *G*_*i*_ still at risk of influenza infection at time *t*. Because we have placed all persons totally immune to infection in group *N* with frailty *b*_*N*_ = 0, we can assume that everyone in groups with nonzero frailty is susceptible at the start of flu season, that is, 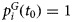 if *b*_*i*_ >0.

The proportion at risk in group *G* as a whole is
1
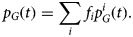


For each subgroup *i*, rate of change with time is
2
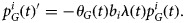


We define the mean frailty among those still at risk in group *G* as
3
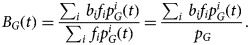


If a proportion *a* of all cases of infection is ascertained (i.e. symptomatic and comes for testing and tests positive for influenza), then the rate at which influenza cases in group *G* present for care and test positive for influenza is
4
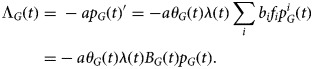


We note that the proportion of the population at risk in each group at time *t*, which we call *p*_*G*_(*t*), will in general differ from the proportion the investigators believe to be at risk in that group, as long as not all cases are ascertained [9]. The proportion thought to be no longer at risk will be the cumulative number infected, times the probability of ascertainment given infection, so the proportion thought at risk will be the complement of that:
5
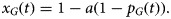


## Results

We present two claims here and give proofs in the appendix. The first claim formalises the intuition described above that, on any day during the influenza season, the direction of bias in estimates of waning will depend on whether depletion-of-susceptibles is greater in the late-vaccinated group (because members became infected before the date of vaccination) or in the early-vaccinated group (because members have already experienced waning by that date and have been exposed to influenza with reduced protection). The second claim considers in turn the four scenarios described in Table 1: with and without true waning, and with and without vaccination complete by the start of influenza season.

Suppose that the influenza season begins at time *t*_0_ after which there is a time-dependent influenza hazard of infection *λ*(*s*) ≥ 0 for *s* >*t*_0_. Let the early-vaccinated group be vaccinated at time *t*_E_ and the late-vaccinated at *t*_L_. These may be before or after *t*_0_. We consider various measures of new cases per unit time at some time *t*_1_ >max (*t*_L_, *t*_0_), that is, after both groups are vaccinated and the season has begun. We define accurate estimation of waning to occur when the rate ratio of new cases for early *vs.* late vaccine recipients is equal to the relative susceptibility of early *vs.* late recipients – that is, when it accurately captures the degree of waning. We will claim that departures from accurate estimation of waning (that is, bias) will occur exactly when the cumulative hazard for the highest-frailty subgroup, modified by vaccination, by time *t*_1_ in group E is different from that in group L. This cumulative hazard is for group *G* is given by 
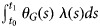
.

In what follows, we refer frequently to ‘overstating’ or ‘understating’ the extent of waning. We define this to mean that an observed rate ratio of cases in the early *vs.* late vaccinees used to estimate waning is respectively larger or smaller than the ratio of actual susceptibilities in the early- *vs.* late-vaccinated groups *θ*_E_(t)/θ_L_(t).

**Claim 1:** When this cumulative hazard at time *t*_1_ is less for the early than the late vaccinees, meaning that
6
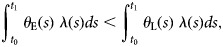


then we will overstate the extent of waning. This will be true for each of three different ways of counting new cases in the two groups.

- The ratio of the raw rate of new cases among early *vs.* late vaccinees will be larger than the ratio of susceptibilities, overstating waning: Λ_E_(t)/Λ_L_(t) >*θ*_E_(*t*)/*θ*_L_(*t*).
- The ratio of the incidence rate of new cases among early *vs.* late vaccinees who are still susceptible to infection will be larger than the ratio of susceptibilities, overstating waning: [Λ_E_(t)/p_E_(t)]/[Λ_L_(t)/p_L_(t)] ≥ θ_E_(t)/θ_L_(t).
- The ratio of the rate of new cases among early *vs.* late vaccinees who are *thought to be* still susceptible to infection (because they have not been observed to have the infection yet) will be larger than the ratio of susceptibilities, overstating waning: [Λ_E_(t)/x_E_(t)]/[Λ_L_(*t*)/*x*_L_(*t*)] ≥ *θ*_E_(*t*)/*θ*_L_(*t*).

Moreover,

1. if inequality (6) is reversed, then inequalities a, b and c are reversed: rate ratios will be less than the true ratio of susceptibilities, and waning will be understated.

Inequality b will be strict if there is heterogeneous frailty (*N* >1). Inequality c will be strict if there is heterogeneous frailty (*N* > 1) and/or imperfect ascertainment of cases (*a* <1), and equal otherwise (*a* = *N* = 1). All inequalities will become equalities if the two sides of Equation (6) are equal.

**Claim 2:** The particular cases considered in Table 1 are true, following from claim 1:

- Top left of Table 1: If there is no waning (so that *θ*_E_(*t*)/*θ*_L_(*t*) = 1 for *t* >*t*_L_) and vaccination is not completed before the start of influenza season (*t*_L_>*t*_0_), then for all for *t* >*t*_L_, the following inequalities will hold, potentially producing erroneous inferences of waning:
  - Λ_E_(t) >Λ_L_(*t*) (early-vaccinated persons will have a higher rate of new cases than late-vaccinated ones).
  - Λ_E_(t)/p_E_ ≥ Λ_L_(*t*)/*p*_L_ (early-vaccinated persons will have a higher incidence rate of new cases *per susceptible* than late-vaccinated ones). Here the inequality is strict if there is heterogeneous frailty, but if frailty is homogeneous (only *N* = 1 subgroup in each group) then equality holds and no waning would be inferred.
  - Λ_E_(t)/x_E_ ≥ Λ_L_(*t*)/*x*_L_ (early-vaccinated persons will have a higher incidence rate of new cases *per person thought to be susceptible* than late-vaccinated ones). Here, the inequality is strict if there is either heterogeneous frailty (*N* >1) or imperfect ascertainment (*a* <1), but equality holds if neither of these applies (*a* = *N* = 1).
- Top right of Table 1: If there is waning and vaccination is not completed before the influenza season, the net bias may go either way. If Equation (6) holds and 
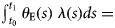
 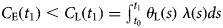, then waning will be overestimated, but if the inequality is switched, it will be underestimated.
- Bottom row of Table 1: If vaccination is completed before influenza season begins (*t*_E_<*t*_L_<*t*_0_), then the following inequalities will hold, with waning underestimated when it exists and correctly estimated as null when it does not.
  - Λ_E_(t)/Λ_L_(t) ⩽ *θ*_E_(*t*)/*θ*_L_(*t*) (the rate ratio of new cases among early-vaccinated persons *vs.* late-vaccinated ones will be less than or equal to the true ratio of susceptibilities) with equality under the null of no waning (when *θ*_E_(*t*)/*θ*_L_(*t*) = 1.)
  - [Λ_E_(t)/p_E_(t)]/[Λ_L_(*t*)/*p*_L_(*t*)] ⩽ *θ*_E_(*t*)/*θ*_L_(*t*) (the incidence rate ratio *per susceptible* will be less than or equal to the true ratio of susceptibilities) with equality under the null of no waning or when frailty is homogeneous (*N* = 1).
  - [Λ_E_(t)/x_E_(t)]/[Λ_L_(*t*)/*x*_L_(*t*)] ⩽ *θ*_E_(*t*)/*θ*_L_(*t*) (the incidence rate ratio *per known susceptible* will be less than or equal to the true ratio of susceptibilities) with equality under the null of no waning or when *a* = *N* = 1, i.e. both (i) frailty is homogeneous and (ii) case ascertainment is perfect.

## Discussion

We have formalised and proved in the appendix the claims summarised in Table 1 about the direction of bias when various study designs are employed to assess whether vaccine protection against influenza infection wanes within a season with increasing time since vaccination. If a study compares the incidence of influenza among persons with early *vs.* late vaccination, and if all vaccinations are completed before the start of influenza season, the design will be unbiased under the null: no waning will be inferred. Under the alternative hypothesis that waning does occur, its extent will be underestimated. Therefore, if waning is inferred, the inference that it is occurring is robust, and the true magnitude may be larger than what is inferred. It is theoretically possible that, if true waning occurs, early vaccination could even look more protective than late when comparing instantaneous incidence (a massive understatement of waning, sufficient to change the sign of the effect) because of the phenomenon of crossing hazards [4, 12], but at least a signal of waning cannot occur spuriously due to the depletion-of-susceptibles bias, if all vaccination is complete by the start of the season.

On the other hand, in a design where some vaccinations occur after the start of influenza season, the estimate is biased under the null: if there is no waning of vaccine-induced protection, waning will be inferred spuriously. If there is waning, the direction of bias is more difficult to determine (see claim 1).

The demonstrations of each of our findings for the vaccinee-only design rely on the same principle, applied differently when the timing of vaccination relative to the season is different. The common principle is that a group that has more vaccine-induced protection will retain a higher proportion of susceptible or highly susceptible individuals, while these will be depleted faster in the group with less vaccine-induced protection. The investigators will be unable to track this differential depletion if (1) susceptibility (frailty as we called it in line with other literature) is variable but unmeasured and/or (2) infections are not all ascertained (e.g. due to some being mild or asymptomatic), so the population at risk is less than that thought to be at risk, especially in the less-protected group.

This common principle is applied in opposite ways in different scenarios, because the late-vaccinated group is more depleted when some influenza incidence occurs before they are vaccinated, and the early-vaccinated group is more depleted when protection wanes. In claim 1, we show how these alternative directions of bias balance when both are present, with bias towards less waning if the effect of waning dominates, and bias towards more waning if the depletion-of-susceptibles from the late-vaccinated group before they received vaccine dominates. In claim 2, we apply this to particular cases and mathematically confirm previous heuristic results – that waning estimates would be null when there is really no waning if vaccination is complete before influenza circulation, that waning would be underestimated if it truly exists and vaccination is complete before influenza circulation, and that waning will be erroneously inferred if it does not exist if vaccination is incomplete at the start of the influenza season. These lead to the recommendation to restrict waning studies to persons vaccinated before influenza season begins.

Estimation biases occurring due to cohort-selection, differential depletion-of-susceptibles or unaccounted-for frailty heterogeneity (three terms for the same phenomenon [4, 6, 8, 9, 12, 13]) have been recognised in the literature for some decades but are often not accounted for in study design and analysis. The analysis here contributes three aspects to the discussion. First, it mathematically separates out the effect of heterogeneous frailty (variation in *b*_*i*_ in our notation, emphasised e.g. in [7, 8, 10]), which leads to the less-protected group being more rapidly depleted of its most frail members and thus looking less at-risk in the aggregate, from the effect of having unobserved infections (more of these in the less protected groups) that deplete the number of persons at any risk differentially from different groups, emphasised for example in [4, 9]. These biases work in the same direction, so that the biases discussed here arise when either or both are present. The second contribution is to show a general condition under which biases in one direction or the other are dominant in a comparison of persons vaccinated on two dates, depending on which group has been more depleted of susceptibles. The third is to show in general that, as proposed in [10], designs that restrict comparison to times of vaccination before the onset of disease exposure are not susceptible to spurious inference of waning. While not applicable for all infections [7], this may be achieved conveniently in highly seasonal diseases where a vaccine can be delivered before transmission begins – such as influenza in temperate climates. The existence of a clear seasonality and the restriction of VE studies to a single season (because vaccine composition and strain circulation may change from season to season) are particular to influenza, so future work could consider extensions to other diseases where vaccine waning may occur over longer periods.

We note that this analysis considers only the biases that result from susceptible depletion (which can be seen as a form of selection bias [14]). It does not consider misclassification that can arise due to imperfect diagnostic tests (in randomised or observational studies), nor does it consider the subtleties that occur if there are multiple infections of the same person in the same year that provide partial and temporary cross-immunity, either from cross-subtype influenza infections. It does not consider other issues of confounding and selection bias, that can plague observational studies in this area [15, 16]. Therefore, it is notable that the concerns about depletion-of-susceptibles bias concern apply even in randomised trials; the reason can be clearly seen, in that the biases occur due to post-randomisation differences that arise between the two arms and influence the outcome (incidence). The exact degree of the bias depends on details of the study design, however. We showed that a bias in the same direction occurs for each of three incidence measures. The first (daily rate of reported cases, without reference to a population at risk) would be most relevant to the classic test-negative case-control design, where no explicit cohort is followed (so depletion-of-susceptibles is entirely unobserved) but rather, incidence of ‘test-negative’ infections is used to assess the population at risk indirectly. The last (rate of reported cases, relative to a population at risk that has been reduced when cases are observed (since by assumption no one can get influenza twice in a season)) is most relevant to a randomised controlled trial or a study similar to that of [3], where a cohort is followed, and persons receiving an influenza diagnosis are removed from the at-risk group (this particular study also removed those who received an influenza test and were negative, but this does not change the general finding). The middle incidence measure would be a target for estimation in a study where every influenza case would be diagnosed and removed from the at-risk group [9]. We considered this to make explicit that, even if this is accomplished (e.g. by virologic or serologic testing) the existence of variable frailty will still lead to the bias. Only if frailty is homogeneous and all infections are perfectly ascertained (or if the vaccine is entirely ineffective, perhaps due to a mismatch) does it completely disappear in general [9]. In the special case where there is no waning, however, the design with preseason vaccination only will be unbiased, and if there is waning, the preseason vaccination design will not overestimate its extent. Therefore a finding of waning under that design (as in [10]), is compelling (unless other important biases are posited), while a failure to detect waning with that design is harder to interpret.

We have described the direction of bias expected under various conditions, but have not quantified its magnitude, which is challenging as many of the underlying parameters, particularly heterogeneous frailty, are not well measured. Qualitatively, the magnitude of the bias can grow if there is greater heterogeneity in frailties, if cumulative incidence is high at the time of comparing early to late vaccinees (leading to greater differences in susceptible depletion), and if VE is high (in a study comparing vaccinees with the unvaccinated) or wanes markedly (in a study comparing early to late vaccinees). For example, in a hypothetical randomised control trial on the timing of influenza vaccination, comparing August vaccinees with November vaccinees after influenza starts circulating in December, in a scenario where

- the true relative hazard ratio is 0.50 for November-*vs.*-August vaccination,
- there are two levels of frailty such that the higher level multiplies risk by 10 and
- 50% of the cohort is highly frail,

if cumulative incidence reaches 9% there would be 2% bias towards the null in the hazard ratio estimate, and if cumulative incidence reaches 45% the bias in the hazard ratio (HR) estimate would be 20% (from 0.50 to 0.60).

The direction of the bias is more likely to be towards understating waning if significant waning occurs between the date of early vaccination and late vaccination, and if there has been little or no incidence by the time of late vaccination, while it is more likely to be towards overstating waning if there has been significant incidence by the time of late vaccination. This is why confining analysis to times of vaccination before appreciable influenza incidence forces the bias to be either zero (if there is no waning) or towards understatement of waning (if there is).

We do have one case where the same data were analysed two ways: first, considering all vaccination dates [3] and again restricting consideration to those who were vaccinated before influenza season [10]. While the latter analysis ensured that if anything waning would be understated according to our framework, it found approximately the same estimate as the earlier analysis, with in fact a slightly higher point estimate and broadly overlapping confidence bounds (18% per month [10] *vs.* 16% per month [3]). For this analysis, it seems the depletion-of-susceptibles bias was small and/or overcome by chance or other factors yielding a slightly larger estimate of waning in the corrected analysis. How this would generalise to other populations is difficult to predict.

In summary, we have provided evidence that a small modification to some existing studies of vaccine waning – specifically, restricting consideration to those vaccinated before influenza season – may be sufficient to make findings of measurable waning very convincing and worthy of consideration in recommendations for the timing of vaccination. We recommend such an approach in future studies, whether experimental or observational.

## Appendix A

### A useful result applied in the main proof

#### Generalised Grönwall inequality

Suppose we have two functions *f*(*t*), *g*(*t*) that are solutions to the following ordinary differential equations (ODEs):

If *f*(0) ≥ *g*(0) >0, then that *f*(*t*) ≥ *g*(*t*) for *t* > 0 as long as

In particular, that holds if *u*(*s*) ≥ *v*(*s*).

Moreover, the inequality *f*(*t*) ≥ *g*(*t*) is strict if either *f*(0) >*g*(0) or .

**Proof:** The ODE for *f*(*t*) can be re-written as *f*^′^/*f* = *u*, which mean (*d*/*ds*)(ln(*f*(*s*)) = *u*(*s*). Integrating this from *t*_0_ to *t* we get

Similarly,

From this the generalised Grönwall equality follows.

## Appendix B

### Proofs of the main claims

**Proof of claim 1:**

By Equation (4), . We prove here that when Equation (6) is true,

B.1

and

B.2

Together these demonstrate claim 1a, and Equation (B.2) alone demonstrates claim 1b.

Proof that Equation (6) implies Equation (B.1):

For each *i*, Equation (6) implies

B.3

Using Equation (3), let , , *v*(*t*) = −*θ*_L_(*t*)*b*_*i*_*λ*(*t*), *u*(*t*) = −*θ*_E_(*t*)*b*_*i*_*λ*(*t*) in the notation of the generalised Grönwall inequality. Equation (B.3) then satisfies the condition of the generalised Grönwall inequality. This implies that , for all *i* and thus by Equation (1) that *p*_E_(t_1_) >*p*_L_(*t*_1_). This is Equation (B.1). QED

Proof of Equation (B.2) when Equation (6) holds:

Assume there are at least two subgroups with different frailties: for subgroups *i* and *j*, with *i* <*j*, we will have *b*_*i*_ >*b*_*j*_. Then Equation (6) implies Equation (B.3), which implies for these two subgroups:

B.4

Now, to show that *B*_E_(t_1_) <*B*_L_(*t*_1_), we need to prove that at time *t*_1_:

Subtracting the LHS from the RHS, we get

Recall that we ordered *b*_*k*_ in the descending order so that *b*_*i*_ >*b*_*j*_ when *i* <*j*. We need, for *i* <*j*, and *t* >*t*_0_ to show that

B.5

We note that the two sides of Equation (B.5) are equal at *t* = *t*_0_.

Differentiating the function  and using the generalised Grönwall inequality, we note that this function is a solution to the ODE:

B.6

Similarly, the function  is a solution to the ODE:

B.7

Thus Equation (B.2) will hold when

but this is Equation (B.4), so we have proven that Equation (6) implies Equation (B.2).

Note that the foregoing relied on heterogeneous frailty (more than one group with different values of *b*_*i*_). When there is one level of frailty (*N* = 1, *b*_1_ = 1), *B*_*G*_(*t*) = 1 for all *G*, *t*.

Having proven Equations (B.1) and (B.2) we have claim 1(a), with always a strict inequality. Having proven Equation (B.2) alone we have claim 1(b). The inequality is strict when there is more than one subgroup with different frailties; otherwise, we have equality.

**Proof of claim 1(c):** To show that , when Equation (6) holds, we note that we have proven  and  when Equation (6) holds. But . Given that *a* ⩽ 1 and *p*_E_ > *p*_L_ ∈ (0, 1], a little algebra shows that that  with equality when *a* = 1. Thus  with strict inequality either *a* <1 (imperfect ascertainment, making the *x*_*G*_ inequality strict) or *N* >1 (heterogeneous frailty, making the *B*_*G*_ inequality strict). We have equality for claim 1(c) when *a* = *N* = 1.

**Proof of claim 1(d):** All of the foregoing proofs are symmetric in groups E and L. If  then results 1(a)–(c) hold with the inequalities reversed, proven by identical arguments. Likewise, if the two sides are equal, then all quantities in the proofs will be equal between groups and claims 1a–c will be show equality.

**Proof of claim 2(i):** If there is no waning (so that *θ*_E_(*t*)/*θ*_L_(*t*) = 1 for *t* >*t*_L_) and vaccination is not completed before the start of influenza season (*t*_L_>*t*_0_), then  because group E will experience protection (*θ*_E_(s) <1 = *θ*_L_(*s*)) for the time between the start of the season or vaccination in group E (whichever is latest), and vaccination of group L (max(*t*_0_, *t*_E_) <*s* <*t*_L_), and thereafter . Therefore the condition of claim 1 is fulfilled, so

by claim 1,

In the case of (b), there is equality when frailty is homogeneous, and the inequality is strict when there is heterogeneous frailty (more than one group with different values of *b*_*i*_), as noted in claim 1(b). In the case of (c), there is equality when ascertainment is perfect and frailty is homogeneous (*a* = *N* = 1), and strict inequality otherwise.

**Proof of claim 2(ii)** (Top right of Table 1): If vaccination is incomplete at the start of the influenza season and waning occurs, then there will be conflicting biases due to depletion-of-susceptibles. Rearranging Equation (6) we have:

where the first integral on the right is negative due to earlier vaccination of group E, and the second integral is positive due to waning. The balance determines whether the extent of waning will be overestimated or underestimated.

**Proof of claim 2(iii)** (Bottom row of Table 1): If vaccination is completed before influenza season begins (*t*_E_<*t*_L_<*t*_0_), then the following inequalities will hold, with waning underestimated when it exists and correctly estimated as null when it does not. This comes from an application of claim 1, with the sign reversed (if there is waning) or equality (if there is no waning). If vaccination is complete before influenza season, then the only source of differences in  is waning; otherwise the cumulative vaccine-adjusted incidence will be equal between groups throughout the study, which will give . Therefore the condition of claim 1 is satisfied (with the inequality reversed) if there is waning, and equality holds in the condition of claim 1 under the null of no waning. From this it immediately follows that:

- with equality under the null of no waning .
- with equality under the null of no waning or when frailty is homogeneous (*N* = 1).
- with equality under the null of no waning or when *a* = *N* = 1, i.e. both (i) frailty is homogeneous and (ii) case ascertainment is perfect.

## References

[1] Puig-Barbera J (2017) Waning protection of influenza vaccination during four influenza seasons, 2011/2012 to 2014/2015. Vaccine 35, 5799–5807. 28941618 10.1016/j.vaccine.2017.09.035

[2] Ferdinands JM (2017) Intraseason waning of influenza vaccine protection: evidence from the US Influenza Vaccine Effectiveness Network, 2011–12 through 2014–15. Clinical Infectious Diseases 64, 544–550. 28039340 10.1093/cid/ciw816

[3] Ray GT (2019) Intraseason waning of influenza vaccine effectiveness. Clinical Infectious Diseases 68, 1623–1630. 30204855 10.1093/cid/ciy770 PMC7182205

[4] Lewnard JA (2018) Measurement of vaccine direct effects under the test-negative design. American Journal of Epidemiology 187, 2686–2697. 30099505 10.1093/aje/kwy163 PMC6269249

[5] Lipsitch M (2019) Challenges of vaccine effectiveness and waning studies. Clinical Infectious Diseases 68, 1631–1633. 30204853 10.1093/cid/ciy773 PMC6495011

[6] Vaupel JW and Yashin AI (1985) Heterogeneity's ruses: some surprising effects of selection on population dynamics. The American Statistician 39, 176–185. 12267300

[7] O'Hagan JJ (2012) Apparent declining efficacy in randomized trials: examples of the Thai RV144 HIV vaccine and South African CAPRISA 004 microbicide trials. AIDS (London, England) 26, 123–126. 10.1097/QAD.0b013e32834e1ce7 PMC3319457 22045345

[8] Halloran ME, Longini IM Jr and Struchiner CJ (1996) Estimability and interpretation of vaccine efficacy using frailty mixing models. American Journal of Epidemiology 144, 83–97. 8659489 10.1093/oxfordjournals.aje.a008858

[9] Kahn R (2019) Analyzing vaccine trials in epidemics with mild and asymptomatic infection. American Journal of Epidemiology 188, 467–474. 30329134 10.1093/aje/kwy239 PMC6357804

[10] Ray GT (2019) Depletion of susceptibles bias in analyses of intra-season waning of influenza vaccine effectiveness. Clinical Infectious Diseases, ciz706. doi: 10.1093/cid/ciz706 [epub ahead of print]. 31351439 PMC7318775

[11] Klein NP and Fireman B (2019) If influenza vaccines wane can we delay vaccination without compromising coverage? Clinical Infectious Diseases, ciz459. doi: 10.1093/cid/ciz459 [epub ahead of print]. 31257408 PMC7346755

[12] Hernan MA (2010) The hazards of hazard ratios. Epidemiology 21, 13–15. 20010207 10.1097/EDE.0b013e3181c1ea43 PMC3653612

[13] Gomes MG (2012) How host heterogeneity governs tuberculosis reinfection? Proceedings. Biological sciences/The Royal Society 279, 2473–2478. 10.1098/rspb.2011.2712 PMC3350683 22357260

[14] Hernan MA, Hernandez-Diaz S and Robins JM (2004) A structural approach to selection bias. Epidemiology 15, 615–625. 15308962 10.1097/01.ede.0000135174.63482.43

[15] Lipsitch M, Jha A and Simonsen L (2016) Observational studies and the difficult quest for causality: lessons from vaccine effectiveness and impact studies. International Journal of Epidemiology 45, 2060–2074. 27453361 10.1093/ije/dyw124 PMC5841615

[16] Sullivan SG, Tchetgen Tchetgen EJ and Cowling BJ (2016) Theoretical basis of the test-negative study design for assessment of influenza vaccine effectiveness. American Journal of Epidemiology 184, 345–353. 27587721 10.1093/aje/kww064 PMC5013887

